# Taming PH_3_: State of the Art and Future Directions in Synthesis

**DOI:** 10.1021/jacs.2c07688

**Published:** 2022-09-07

**Authors:** Thomas M. Hood, Samantha Lau, Ruth L. Webster

**Affiliations:** Department of Chemistry, University of Bath, Claverton Down, Bath BA2 7AY, United Kingdom

## Abstract

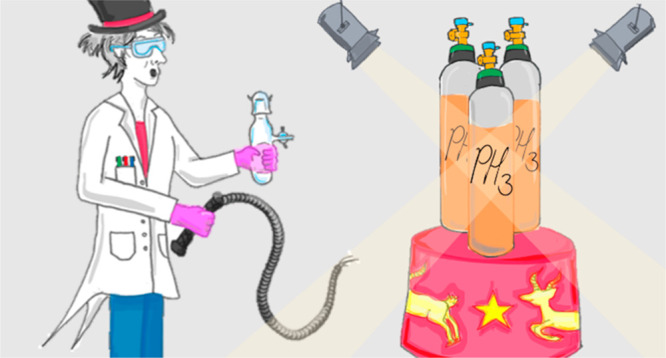

Appetite for reactions involving PH_3_ has grown in the past few years. This in part is due to the ability to generate PH_3_ cleanly and safely via digestion of cheap metal phosphides with acids, thus avoiding pressurized cylinders and specialized equipment. In this perspective we highlight current trends in forming new P–C/P–OC bonds with PH_3_ and discuss the challenges involved with selectivity and product separation encumbering these reactions. We highlight the reactivity of PH_3_ with main group reagents, building on the early pioneering work with transition metal complexes and PH_3_. Additionally, we highlight the recent renewal of interest in alkali metal sources of H_2_P^–^ which are proving to be useful synthons for chemistry across the periodic table. Such MPH_2_ sources are being used to generate the desired products in a more controlled fashion and are allowing access to unexplored phosphorus-containing species.

## 1 Introduction

There is an acute need to undertake drastic changes in the way we consume the Earth’s vital and finite resources, with much of this linked to changes needed to policies and practices of governments.^1,2^ However, this should also bring into strong focus our need to undertake sustainable synthesis.^3,4^ With this comes the need to develop new methods with which to undertake novel bond transformations; use reagents that avoid the generation of exogenous waste which requires protracted purification procedures; move away from harmful solvents; use feedstocks that are less activated (or come directly from the source, *e.g*., in the Earth’s crust); and use metals that are abundant (*e.g*., rock-forming metals) both in heterogeneous/homogeneous catalysis and in devices/materials.^5^

**Scheme 1:**
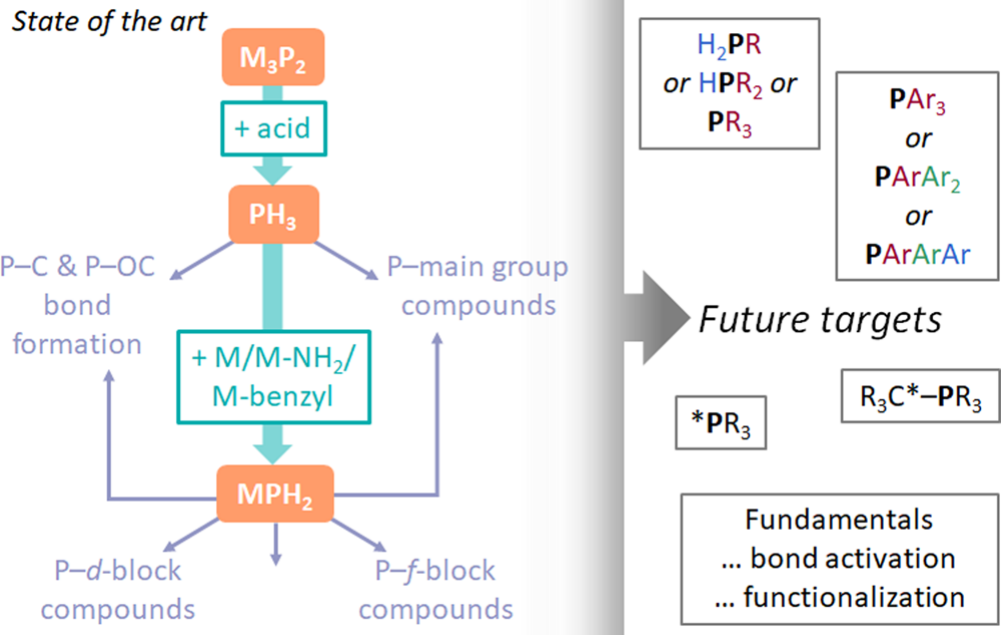
An Overview of the Key Discussion Points Presented in This Perspective

Rather than providing prescriptive coverage of all reports on transformations involving PH_3_, including the pioneering research into stoichiometric reactions with PH_3_,^6^ this perspective serves to highlight trends in the applications of PH_3_, the “routine” P–C bond forming reactions that are base-mediated (*i.e*., reductive coupling) or involve the hydrofunctionalization of unsaturated bonds. This perspective will also cover more unusual transformations that form P–C bonds via other means, along with modern main group bond transformations and reactions with metals (Scheme 1).

The latter portion of this perspective goes beyond PH_3_ and showcases the chemistry of the H_2_P^–^ anion. Recently, the use of alkali metal phosphides as a source of H_2_P^–^ has been receiving renewed interest. We will highlight some of the remarkable implementations of such salts, both in organic transformations and as promising reagents in the preparation of notable main group, transition metal, and f-block metal species.

We would be remiss not to briefly mention the numerous reports, during a prolific period of PH_3_ research that took place between the late 1960s through to the early 1990s, on the reaction between PH_3_ and transition metal (TM) complexes.^7−15^ Formation of TM complexes bearing the [TM]–PH_3_ motif as well as formal oxidative addition/hydride abstraction to form [TM]–PH_2_ and [TM]–(μ-PH_2_) motifs have been identified, with analysis ranging from multinuclear NMR and IR data only, through to those also reporting single crystal X-ray diffraction data. For example, Jones and co-workers reported the formation and isolation of a remarkably air-stable *trans*-[RuCl_2_(PH_3_)_4_] complex.^16^ However, further study into reactivity with these complexes has rarely been explored^17^ and will therefore not be the focus of this perspective. However, this highlights the many seemingly simple areas of PH_3_ research that are yet to be fully investigated.

## 2 An Important Note on Safety

It would be irresponsible not to emphasize the dangers associated with handling PH_3_. PH_3_ is a highly toxic gas that is spontaneously flammable in air. The American Conference of Governmental Industrial Hygienists (ACGIH) places a time-weighted average limit on exposure at 0.05 ppm (which is the concentration of a substance to which most workers can be exposed without adverse effects), with a short-term exposure limit of 0.15 ppm (which means a 15 min time-weighted average exposure should not be exceeded at any time during an 8 h workday).^18^ The National Institute for Occupational Safety and Health (NIOSH) list 0.3 ppm as the time-weighted average limit and 1 ppm as the short-term exposure limit (10 h, 15 min respectively). The US Environmental Protection Agency lists that the 4 h LC_50_ for PH_3_ in rats is 11 ppm.^19^ To put these numbers into context, the NIOSH time-weighted average limit for CO is 35 ppm and the NIOSH short-term exposure limit for HCN is 4.7 ppm,^20^ while the 1 h LC_50_ for HCN in rats is 139–144 ppm.^21,22^ In short, handling of pressurized cylinders of PH_3_ requires a robust risk assessment/COSHH assessment and rigorous safety protocols, not least a PH_3_ monitor to ensure the safety of not only the chemist handling the substance but also other researchers in the lab. The fume hood setup must include NaOCl scrubbers or a PH_3_ burner/H_2_O spray to quench unreacted PH_3_. Akin to our responsibility to study and develop more sustainable approaches to synthesis, safe use and quenching of this toxic gas, avoiding exposure of researchers and the environment to this species, is paramount.

## 3 Reacting PH_3_ To Form P–C/P–OC Bonds

PH_3_ is the next downstream output from elemental phosphorus, which comes directly from industrial large-scale processing of phosphate rock.^23^ Numerous reviews on functionalization of P_4_ exist, but the tetra-nuclear nature of this feedstock means that controlled, direct, or catalytic functionalization of P_4_ into, for example, 4PR_3_ is not well-documented.^24,25^ Instead, conversion of P_4_ into PH_3_ or PCl_3_ and onward reaction to form organophosphines is the more traditional pathway. Onward reactions of PCl_3_ with organic substrates to prepare P–C(*sp*^3^) bonds are well documented, but wasteful in terms of atom economy.

P–C bond forming reactions with PH_3_ range from stoichiometric-in-base-mediated reactions with alkyl halides through to hydrophosphination in the presence of a metal catalyst, radical initiator, or a base. In many cases we invariably access products of the form PR_3_, although there are examples where HPR_2_ and H_2_PR are produced (*vide infra*). Even the hydrophosphination literature has limitations: work on catalytic hydrophosphination has routinely reported on the formation of the tertiary phosphine product as the major species, and only limited progress in diversifying the structure of the products, or the reaction selectivity, has been made. The reason that PR_3_ is formed preferentially can be linked to the enhanced reactivity of the product compared to the starting materials, *i.e*. PH_3_ < H_2_PR < HPR_2_, and accessing H_2_PR or HPR_2_ tends to be achieved by limiting substrate stoichiometry rather than any greater form of reaction control. Stoichiometric-in-base transformations are simple to undertake and are well documented, but it could be argued that they serve to demonstrate the limitations in the organic transformations undertaken using PH_3_: the reaction of RCl + base + PH_3_ is simply the inverse of the classical method of using RH + base (or RCl + 2base) + PCl_3_.

**Scheme 2:**
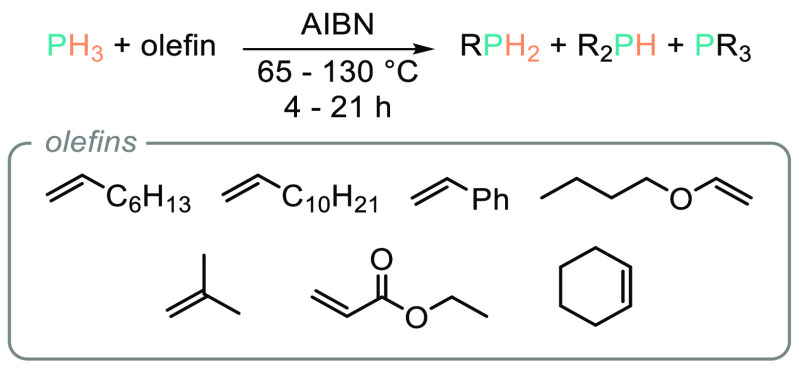
Rauhut and Co-workers’ Early Study into Radical Mediated Hydrophosphination of Activated and Unactivated Alkenes

PH_3_ is a reactive substrate, and the early work on the formation of phosphonium salts from PH_3_ and formaldehyde dates back to at least 1888,^26^ with applications from this seminal study still very relevant today.^27^ Building upon work from Stiles et al. using photochemical initiation,^28^ an early report on catalytic functionalization of PH_3_ came from Rauhut and co-workers^29^ where they disclosed the hydrophosphination of acrylonitrile using PH_3_ and aqueous KOH at room temperature. The reaction is mild, but is somewhat lacking in control, producing mixtures of primary, secondary, and tertiary cyanoethyl phosphines. Excess acrylonitrile allowed the formation of the tris-substituted product in 80% yield, and the secondary product could be formed in 58–63% when an excess of PH_3_ is employed, while the primary cyanoethyl species is formed in 52% yield, but an autoclave operating at high pressure of PH_3_ is needed (28–32 atm). The mono- and bis-substituted products were further employed in radical-mediated hydrophosphination reactions.^30^ Rauhut and co-workers also employed azobis(isobutyronitrile) (AIBN) as a radical initiator to hydrophosphinate with a range of alkenes in the presence of PH_3_.^30^ Interestingly, although the ratio of primary/secondary/tertiary organophosphine product is often in the region of 1:1:1, reactions with unactivated systems such as 1-octene, 1-dodecene, cyclohexene, isobutylene, and butyl vinyl ether are reported (Scheme 2). In fact, 1-octene (1 mol), PH_3_ (0.33 mol), and AIBN (5 mol %) is an exothermic reaction that generated a reaction temperature of 80–100 °C and produced 83% tris(octyl)phosphine cleanly after 6 h; this reaction is furthermore impressive as transformation of unactivated reagents has largely eluded modern hydrophosphination catalysis.^31^

**Scheme 3:**
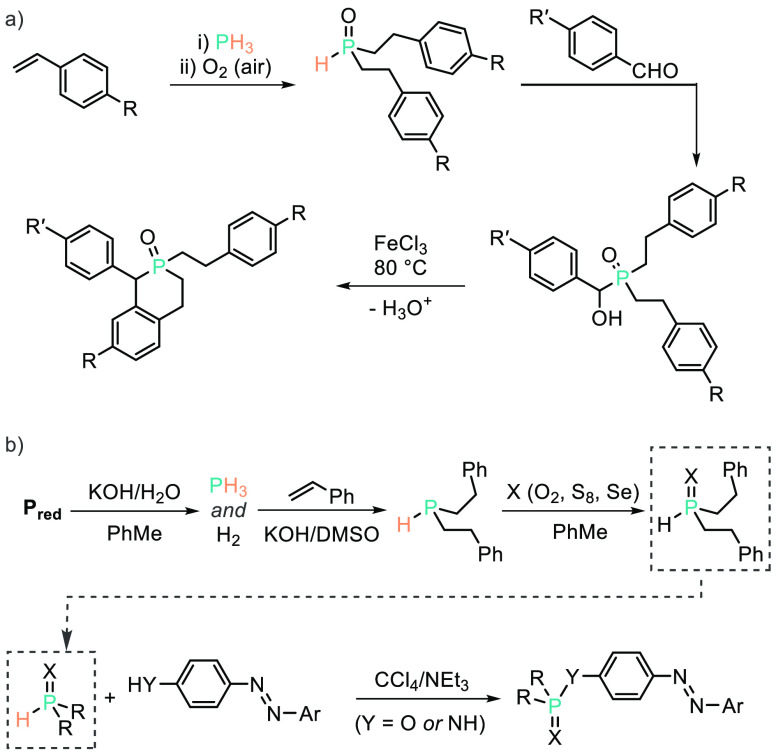
Trofimov Has Employed Methods Similar to Those of Rauhut and Co-workers, But Has Extended This To Prepare (a) Phosphacycles and (b) Functionalized Diazo Compounds

Similar to the earlier work of Rauhut and co-workers, Trofimov and co-workers have recently reported base-mediated hydrophosphination of 2 equiv. of styrene (or 4-*t*Bu-styrene) with PH_3_. The authors have published two possible onward transformations. The first is oxidation, to generate the anti-Markovnikov secondary phosphine oxide product, which is then used as a nucleophile to react with an aldehyde and finally, in the presence of FeCl_3_, hydroxide abstraction to generate a carbocation in an S_N_1-type process. This then allows cyclization to form a phosphinoline oxide product (Scheme 3a).^32^ The second possible onward transformation is P–O or P–N bond formation at the *para*-position of azobenzenes, using a simple base to carry out the coupling reaction (Scheme 3b).^33^ The UV/vis-mediated isomerization of the azo functionality was then investigated. Ragogna employed AIBN to prepare tertiary fluorinated alkyl phosphines which can then be transformed into phosphonium salts to attenuate the properties of UV-curable resins.^34^ Ragogna has also employed the early methods to prepare phosphinated lignin, which is effective as a metal scavenger.^35^

A hydrophosphination that, unsurprisingly, does not need any activating agents or a catalyst is the reaction of PH_3_ with the highly activated imine 1,1,1,3,3,3-hexafluoropropan-2-imine, generating 4.75 g (96% yield) of the geminal substituted NH_2_,PH_2_ product from a large-scale synthesis.^36^

**Scheme 4:**
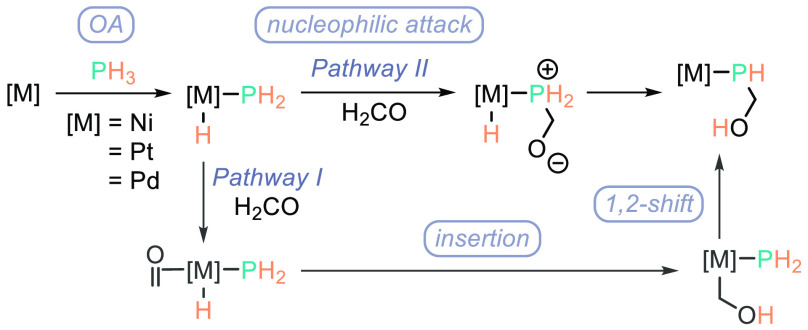
Pringle’s Postulated Mechanism for the Hydrophosphination of Formaldehyde

In contrast, many hydrophosphination reactions involving PH_3_ have employed transition metal catalysts; Pringle undertook the seminal work in this field and used platinum chloride salts, as well as tetrakis(phosphino) Pt, Pd, and Ni complexes for the reaction of formaldehyde with PH_3_.^37−39^ Pringle also reported the use of [Pt(norbornene)_3_] as an effective precatalyst for the reaction of PH_3_ with ethyl acrylate.^40^ Finally, a series of tris(phosphino) Ni, Pd, Pt catalysts as well as tris(phosphino) iridium chloride complexes were reported as competent catalysts for the hydrophosphination of acrylonitrile.^41,42^ For all three unsaturated substrates the tertiary PR_3_ product is formed as the sole product, although a mixture of products is often observed *in situ* due to the stepwise nature of the reactions. A generic catalytic cycle involves coordination of PH_3_ with the unsaturated M(0) center, oxidative addition (OA) to generate a mixed metal(II) hydrido phosphide species, and then insertion of the unsaturated bond into the M–H bond followed by a reductive 1,2-shift step to generate the M–PR_3_ product. An alternative pathway for formaldehyde involves a nucleophilic attack on the carbonyl moiety by M–PH_2_ forming a zwitterion, and then hydride transfer generates the M–PR_3_ product (Scheme 4).

More recently Trifonov reported the use of 1,3-diisopropylimidazol-2-ylidene and 1,3-diisopropyl-4,5-dimethylimidazol-2-ylidene as well as their complexes [(Me_3_Si)_2_N]_2_M(NHC)_2_] (M = Ca, Yb, Sm) as precatalysts for hydrophosphination with PH_3_. Remarkably, they report the generation of primary phosphines based on the feedstock stoichiometry.^43^ A particularly intriguing reaction from this publication is the formation of tri(*Z*-styryl)phosphane; the acidic nature of both the phenylacetylene and PH_3_ along with the selectivity for the kinetic all-*Z* product is remarkable. Transformations of this type warrant further investigation in terms of substrate scope (and with this *E*/*Z* selectivity) and onward functionalization with an eye toward applications.

**Scheme 5:**
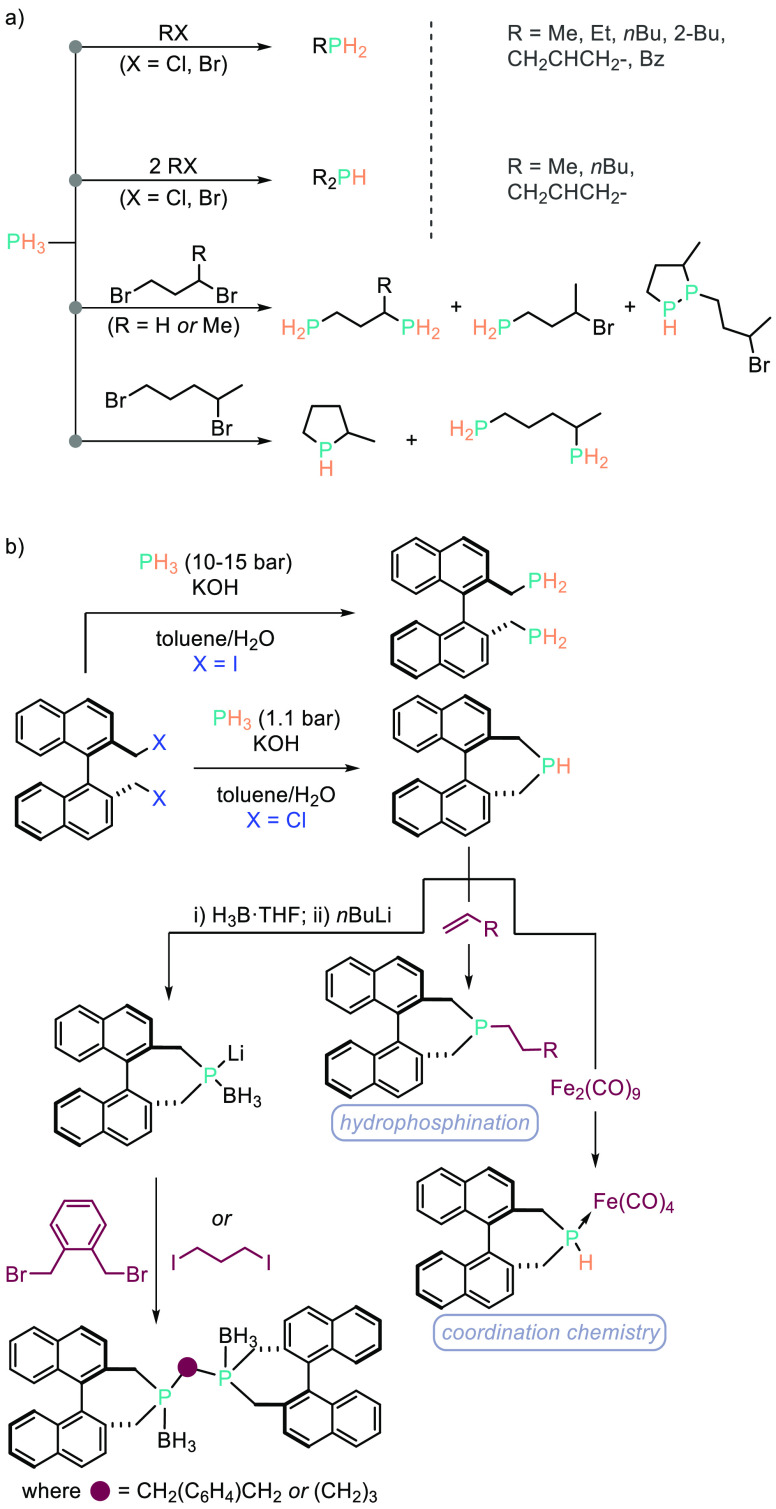
Stelzer and Co-workers Have Prepared a Wealth of Different Phosphorus Compounds (a) Using a Base and/or Phase-Transfer Conditions and (b) Employing These Methods To Prepare 1,1′-BINAP-Derivatives from PH_3_

Stoichiometric transformations are prevalent in the literature and follow similar trends in terms of the products formed and the makeup of the transformation. For example, Stelzer and Sheldrick report a KO*t*Bu route to prepare water-soluble phosphindoles/phosphindole oxides from PH_3_.^44^ Stelzer has also reported on exploiting the inherent equilibrium between PH_3_ + OH^–^ ⇌ H_2_P^–^ + H_2_O when aqueous DMSO/KOH solution (or with the inclusion of phase transfer catalysts such as (*n*Bu)_4_NCl) is used, thus allowing generation of low concentrations of the highly nucleophilic H_2_P^–^ ion for the selective reaction with organohalides forming (stoichiometry-driven) primary or secondary alkyl phosphines, bis(alkyl)phosphines, and cyclic phosphines (Scheme 5a).^45^ Stelzer and co-workers have also employed iodine to prepare structurally exciting PH_2_–BINAP (1,1′-binaphthyl) and PH–BINAP systems (Scheme 5b).^46^

**Scheme 6:**
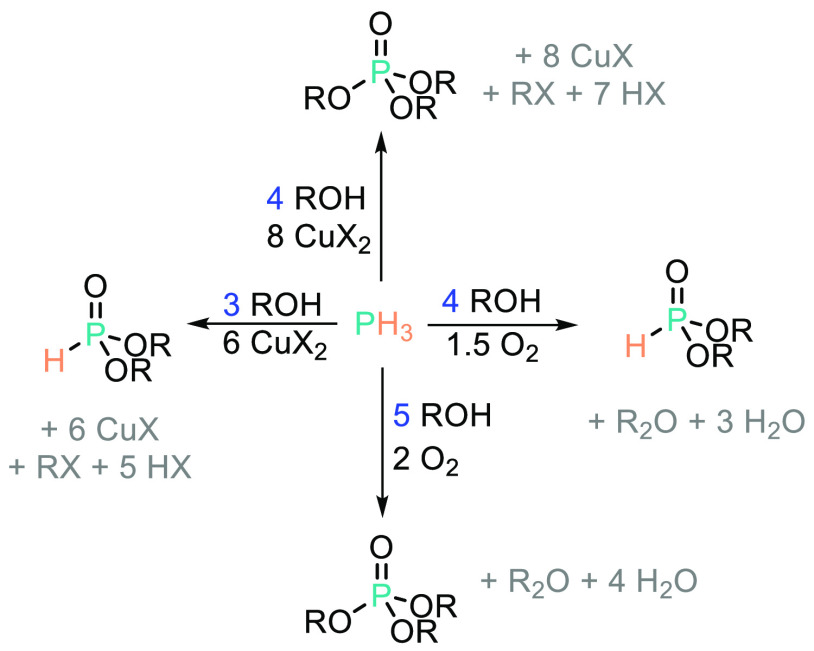
Borangazieva and Co-workers Have Showed That Reagent Stoichiometry Can Be Used to Influence the Product Distribution When Preparing P–OR Bonds

Borangazieva and co-workers have reported an I_2_/pyridine system for the formation of trialkyl phosphates from PH_3_ and methanol/ethanol/butanol/amyl alcohol/octanol.^47^ This methodology was further extended to the preparation of primary aminoalkyl phosphines.^48^ A change to stoichiometric CuCl_2_ in CCl_4_ gives selective formation of the dialkyl phosphite (HP(O)(O*i*Pr)_2_) when isopropanol is employed (Scheme 6).^49^ Further study into the reaction, and inclusion of quinone as a reductant, has also been reported.^50^

However, in general, secondary and primary species are observed as side products in many reactions. Given the challenges associated with purification of reactive phosphines, particularly those that are the product of hydrophosphination reactions (where the product is invariably an alkyl phosphine and thus more prone to oxidation), selective synthesis of a primary *or* secondary *or* tertiary phosphine is desirable. Moreover, when we look at commercial organophosphines, very few tertiary monophosphines are symmetrically substituted; there are key organophosphines such as PPh_3_, PCy_3_, and P*t*Bu_3_, but organophosphines routinely used in, for example, cross-coupling reactions include SPhos (2-dicyclohexylphosphino-2′,6′-dimethoxybiphenyl) and XPhos (2-dicyclohexylphosphino-2′,4′,6′-triisopropylbiphenyl), and bis(phosphines) such as dppf (1,1′-ferrocenediyl-bis(diphenylphosphine), XantPhos (4,5-bis(diphenylphosphino)-9,9-dimethylxanthene), and dppe (1,2-bis(diphenylphosphino)ethane) to name but a few. Here we raise another issue of a pure atom-economy-driven approach to PH_3_ functionalization: that being that the preparation of P–Ar bonds is limited to work from Dorfman and Levina, and more recently Wolf (*vide infra*).

## 4 PH_3_ Reacting with Compounds of the *p*-block

**Scheme 7:**
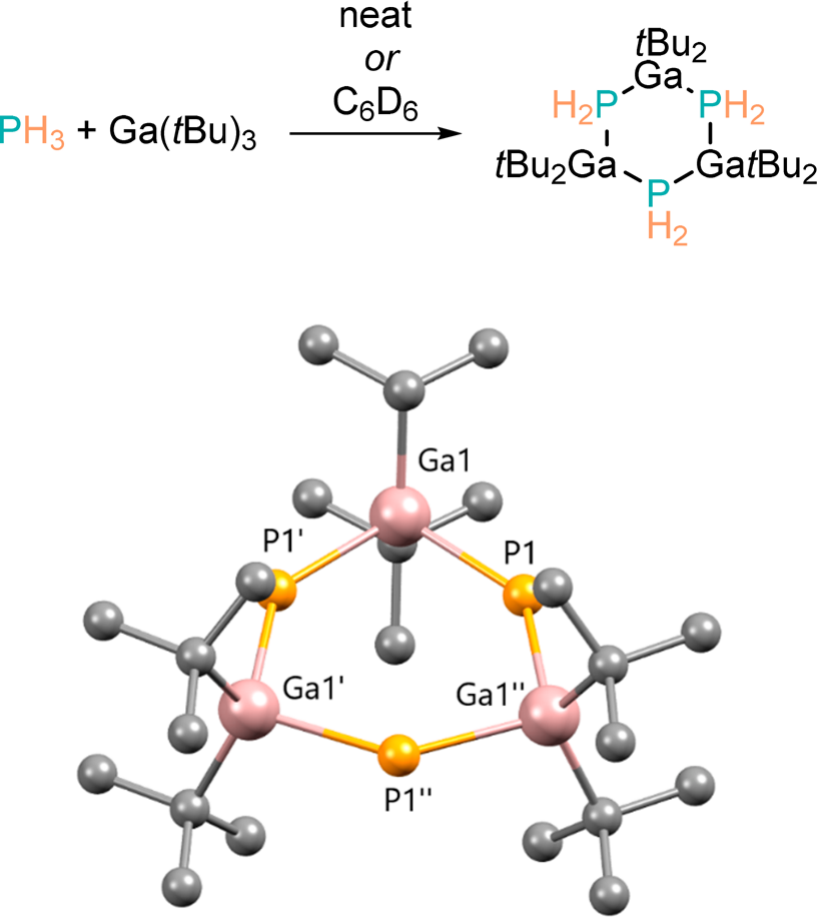
Ga(*t*Bu)_3_ Reacts with PH_3_ To Form Gallium Phosphide Ring Structures The POV-Ray image of the single crystal X-ray structure (CCDC 1197532) shows a distorted 6-membered ring, but this is completely planar with no puckering. All H atoms removed for clarity.

In the 1990s, Cowley and Jones undertook investigations into the reactivity of PH_3_ with an alkyl gallium compound with a view to preparing precursors for OMCVD processes (organometallic chemical vapor deposition). The authors present a highly sensitive μ-PH_2_ cluster which undergoes slow decomposition at 200 °C (Scheme 7).^51^

**Scheme 8:**
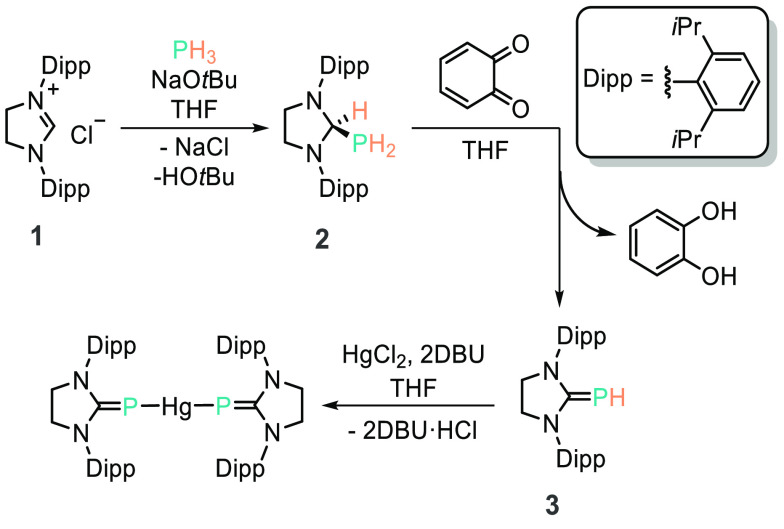
Grützmacher’s Reported Oxidative Addition of PH_3_ by an NHC Which Can Then Form the Phosphinidene, **3**, Driven by the Aromatization of 1,2-Benzoquinone

Further reports on reactions of PH_3_, which we may consider being in the realm of main group bond transformations, are those involving NHCs and their heavier group 14 congeners. Grützmacher and Pringle reported the *in situ* generation of the SIPr (1,3-bis(2,6-diisopropylphenyl)imidazolidine-2-ylidene) NHC (**1**, Scheme 8) from the chloride salt, which forms the PH_2_-imidazolidine product (**2**) from reaction with PH_3_ and base (or the *tert*-butoxide adduct of NaPH_2_). This product can then undergo hydrogen abstraction, driven by the aromatization of *ortho*-quinone, which allows the formation of the formal phosphinidine-carbene adduct (**3**).^52^ This latter species was shown to undergo complexation with HgCl_2_.

**Scheme 9:**
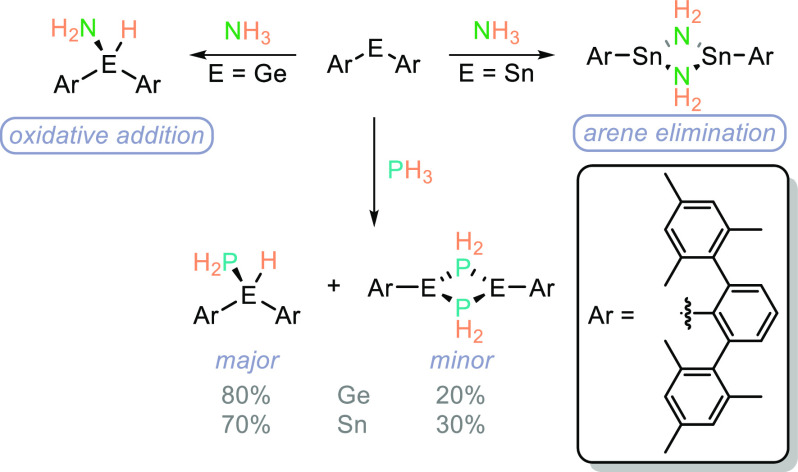
Ragogna and Power Have Demonstrated the Divergent Reactivity of NH_3_ and PH_3_ That Is Observed in the Presence of Group 14 Tetrylenes

Ragogna and Power have shown that PH_3_ can oxidatively add to tetrylenes. The authors note a divergence in reactivity when comparing the chemistry of NH_3_^53−56^ and PH_3_; the former gives the OA product with Ge and the arene elimination dimeric product with the Sn congener (Scheme 9). However, when PH_3_ is employed a mixture of the OA and arene elimination dimer is formed with both Ge and Sn (the OA product is the major species in both cases).^57^ Similar to the work of Ragogna and Power, where there is a discrepancy between the reactivity of the lighter and heavier pnictogens, Driess has demonstrated a difference in reactivity of PH_3_ compared to AsH_3_ when undertaking OA to silylene compounds.^58^ PH_3_ generates the OA product, whereas with AsH_3_, although OA takes place, there is an equilibrium between the arsenide product and the isomerized arsinidine species, making use of the ligand system to invoke this process.

**Scheme 10:**
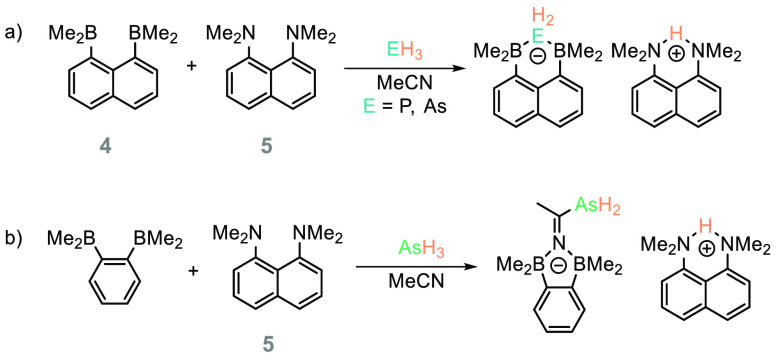
(a) Proton and Hydride Sponge Have Been Used To Activate PH_3_ and AsH_3_; (b) with 1,2-Bis(dimethylboranyl)benzene, the Reactivity of PH_3_ Is Unchanged (Not Shown), but AsH_3_ Reacts with Concomitant MeCN Functionalization

Mitzel has employed both hydride sponge (**4**) and proton sponge (**5**, Scheme 10) as a frustrated Lewis pair (FLP) system to activate a range of small molecules, including PH_3_.^59^**5** undertakes proton abstraction while **4** forms the phosphide adduct, and the authors note that QTAIM (quantum theory of atoms in molecules) analysis indicated that the B–P bond interaction is the most covalent B–E bond interaction when compared to the N, As, O, S, and Se analogues in the study. Interestingly, when the hydride sponge is modified, although PH_3_ undergoes the same activation event, AsH_3_ undergoes a further transformation with the MeCN solvent. If we consider the wealth of transformations that can be undertaken both stoichiometrically and catalytically using FLPs,^60−63^ in particular reactions that use H_2_ that has been activated,^64,65^ this hints that this could be a rich vein of research. Indeed, modification of the FLP structure could enable enantioselective transfer of the H_2_P^–^ and H^+^ fragments to an organic substrate.

## 5 Future Targets

At this stage it is useful to consider several aspects as we look toward future synthetic development targets with PH_3_ or MPH_2_. In an atom-economic, chemoselective manner, with wide-ranging functional group tolerance, key targets should include the following:

1. Controlled synthesis of primary *or* secondary *or* tertiary organophosphines. Reactions need to avoid the formation of mixtures that require complicated cleanup procedures or additional reduction steps to access the P(III) species from the P(V) phosphine oxide;
2. The synthesis of unsymmetrically substituted phosphines from PH_3_ or MPH_2_ and ultimately *C*- or *P*-stereogenic phosphines;
3. The preparation of P–Ar phosphines from PH_3_ or MPH_2_, *e.g*. PAr_3_ through to P*Ar^1^Ar^2^Ar^3^ selectively;
4. Unique methods not only to activate PH_3_ but also undertake onward functionalization, *e.g*. chemistry beyond hydrofunctionalization.

However, to make such advances PH_3_ and MPH_2_ needs to become more accessible to a wider range of researchers. Indeed, we envisage that many advances will be possible simply through PH_3_ (or analogues of PH_3_) being used more widely in research.

## 6 Alternative Sources of PH_3_

The industrial standard for the production of PH_3_ is the base-mediated disproportionation of white phosphorus, in the so-called Hoesch process.^66^ P_4_ is treated with sodium or potassium hydroxide at slightly elevated temperatures (50 °C). With very careful conditions the gas can be collected in ∼95% purity, though this procedure is not particularly practical for a research laboratory. The lab-scale synthesis of PH_3_ has been achieved in a number of ways: by the treatment of PCl_3_ with Na metal (followed by hydrolysis),^67^ the high-temperature treatment of black phosphorus in liquid hydrogen,^68^ and the pyrolysis of either hypophosphorous acid, phosphorous acid, or a salt of one of these acids.^69^ In 1967, the pyrolysis of phosphorous acid was described as the “most convenient” method for the generation of PH_3_;^69^ however, in a modern research laboratory the idea of isolating PH_3_ as a liquid by consecutive condensation and distillation is perhaps a barrier to implementation for many researchers. Additionally, Trofimov reported the generation of PH_3_ from red phosphorus by treatment with aqueous KOH at 65–75 °C; however, this reaction is concomitant with the generation of dihydrogen and as such is limited to reactants that are inert toward dihydrogen and moisture.^70,71^

### 6.1 Metal Phosphides for PH_3_ Release

Handling pressurized gases, irrespective of toxicity, requires a level of rigor that is not necessarily required when handling solids. Recent reports on the use of metal phosphides, *e.g*. Zn_3_P_2_, AlP, and Mg_3_P_2_, for the *in situ* release of PH_3_ by digestion using an acid, *e.g*. HCl, provide a route to PH_3_ research that was previously inaccessible to many. However, PH_3_ is still released from the metal phosphide; indeed these phosphides are routinely used as pesticides because of their ability to release PH_3_ on ingestion, which is fatal. Therefore, although easy to obtain, inexpensive (approximately $74 per kg^72^), and easy to handle, the same level of care and safety assessment should be taken when handling these simple salts as handling PH_3_ gas cylinders.

**Scheme 11:**
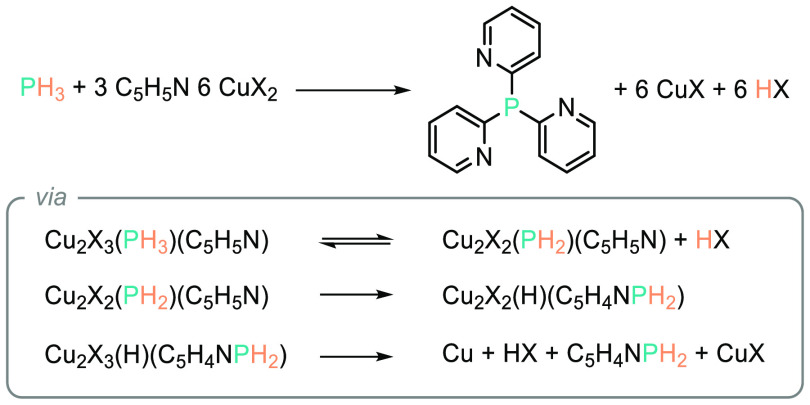
Dorfman Provides a Rare Example of P–Arene Bond Formation Using PH_3_

One of the earliest reports on the *in situ* generation of PH_3_ from a metal phosphide (Zn_3_P_2_) for the preparation of high-value P–C bonds was reported by Dorfman and Levina in 1992.^73^ The authors employ stoichiometric CuCl_2_ or Cu(OAc)_2_, which, in the presence of pyridine in the coordination sphere, is proposed to acidify the P–H bond in PH_3_, forming a putative Cu-phosphide intermediate along with HCl/HOAc. It is postulated that the resonance structure of pyridine is such that it renders the *ortho*- and *para*- positions δ+, and this, coupled with the proximity of the *ortho*-position to the copper center, renders this position prone to attack by H_2_P^–^, generating the tris(pyridin-2-yl)phosphane product selectively (Scheme 11).

**Scheme 12:**
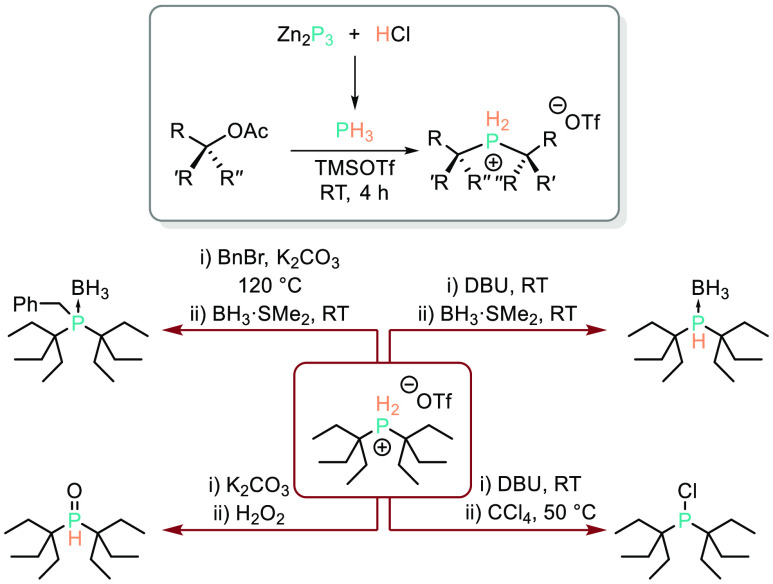
Ball Has Demonstrated That *in Situ* Formation of PH_3_ from Zn_3_P_2_ Can Be Used to Excellent Effect, Furnishing Otherwise Challenging To Access *tert*-Alkyl Phosphines via the Phosphonium Salt

PH_3_ generated by decomposition of Zn_3_P_2_ has been detailed more recently by Ball^74^ and Wolf.^75^ Ball has elegantly demonstrated the application of *in situ* generated PH_3_ for the synthesis of *tert*-alkyl phosphonium triflates, where the byproduct of the reaction is TMSOAc. Due to the high levels of substitution these products cannot be formed using a hydrophosphination route, while the conventional route to secondary alkyl phosphines, R_2_PH, would employ PCl_3_ and organomagnesium or organolithium reagent, followed by reduction of the remaining P–Cl bond with a hydride reagent. Ball has shown that these phosphonium salts can then be converted into the secondary phosphine chloride on reaction with 1,8-diazabicyclo[5.4.0]undec-7-ene (DBU) and CCl_4_, transformed into their BH_3_ protected phosphine congener (using DBU/BH_3_·SMe_2_), benzylated (BnBr then protected using BH_3_·SMe_2_) or oxidized to the phosphine oxide (using K_2_CO_3_ then H_2_O_2_), Scheme 12.

**Scheme 13:**
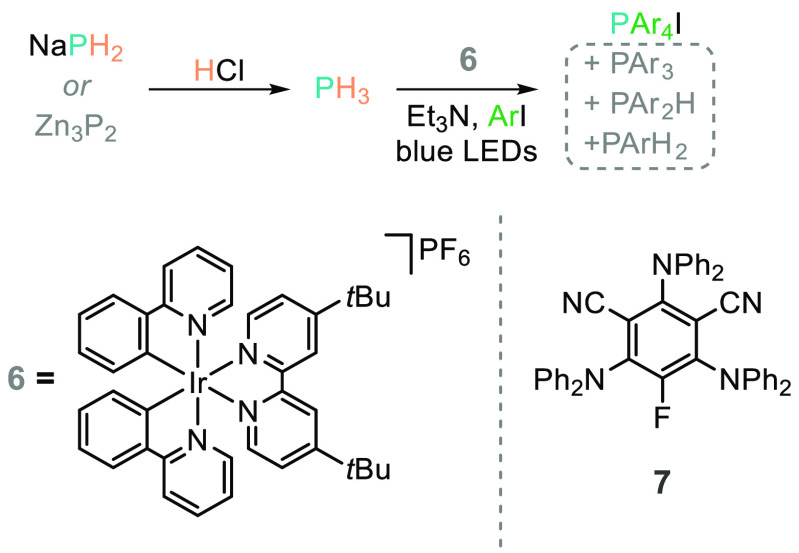
Photocatalysis Has Been Used by Wolf and Co-workers To Prepare Arylphosphines from PH_3_

Using a similar reaction setup to Ball, where Zn_3_P_2_ is digested using HCl in an H-tube and the *in situ* generated PH_3_ can then react with substrate in the second chamber, Wolf employed iridium photocatalyst (**6**) NEt_3_, PhI, and blue LEDs to prepare Ph_4_P^+^I^–^ in a 35% yield after 48 h (Scheme 13). However, use of NaPH_2_ as an alternative to PH_3_ was more successful, generating the product in 77% yield after 24 h. Extending the substrate scope beyond PhI, but continuing to use NaPH_2_, the authors show selective triarylation using sterically encumbered 2-methyliodobenzene (63% Ar_3_P with no other arylation products observed) and 2-methoxyiodobenzene (42% Ar_4_P^+^ observed only). While 4-methyliodobenzene gives 64% Ar_4_P^+^/<5% Ar_3_P, 3-methyliodobenzene gives 61%/6% as Ar_4_P^+^/Ar_3_P and 3-methoxyiodobenzene gives 53%/<5% as Ar_4_P^+^/Ar_3_P. The remaining ArI substrates give less attractive ratios of Ar_4_P^+^/Ar_3_P and/or conversions below 50%. A change to an organophotocatalyst (**7**) can lead to modest adjustments in the ratio/conversion to product(s). The reaction mechanism is postulated to proceed via sequential arylation steps, where a photogenerated Ar^•^ reacts with [P]^•^. Reaction profiling shows a rapid buildup of Ph_2_PH as a major species, along with PhPH_2_, which after 5 h are depleted as the onward reaction of these intermediates takes place, with Ar_4_P^+^ eventually being the dominant product. The reaction requires 2 mol % **6**, 11 equiv of ArI, and 15 equiv of NEt_3_ (or 10 mol % **7**, 13 equiv of ArI, 16 equiv of NEt_3_); clearly elegant but, excitingly, with room for modification and diversification.

### 6.2 *In Situ* PH_3_ Generation from P(OR)_3_

Liptrot and co-workers recently presented a Cu-catalyzed route to generate PH_3_ in 30 min from P(OPh)_3_, using HBpin as a reducing agent. PH_3_ was generated in 89% conversion on a 0.1 mmol scale. The *in situ* generated PH_3_ was then directly implemented in the quantitative catalytic hydrophosphination of phenyl isocyanate in a two-pot procedure.^76^

## 7 The PH_2_ Anion

### 7.1 Reactions of MPH_2_ with *p*-Block Compounds

**Scheme 14:**
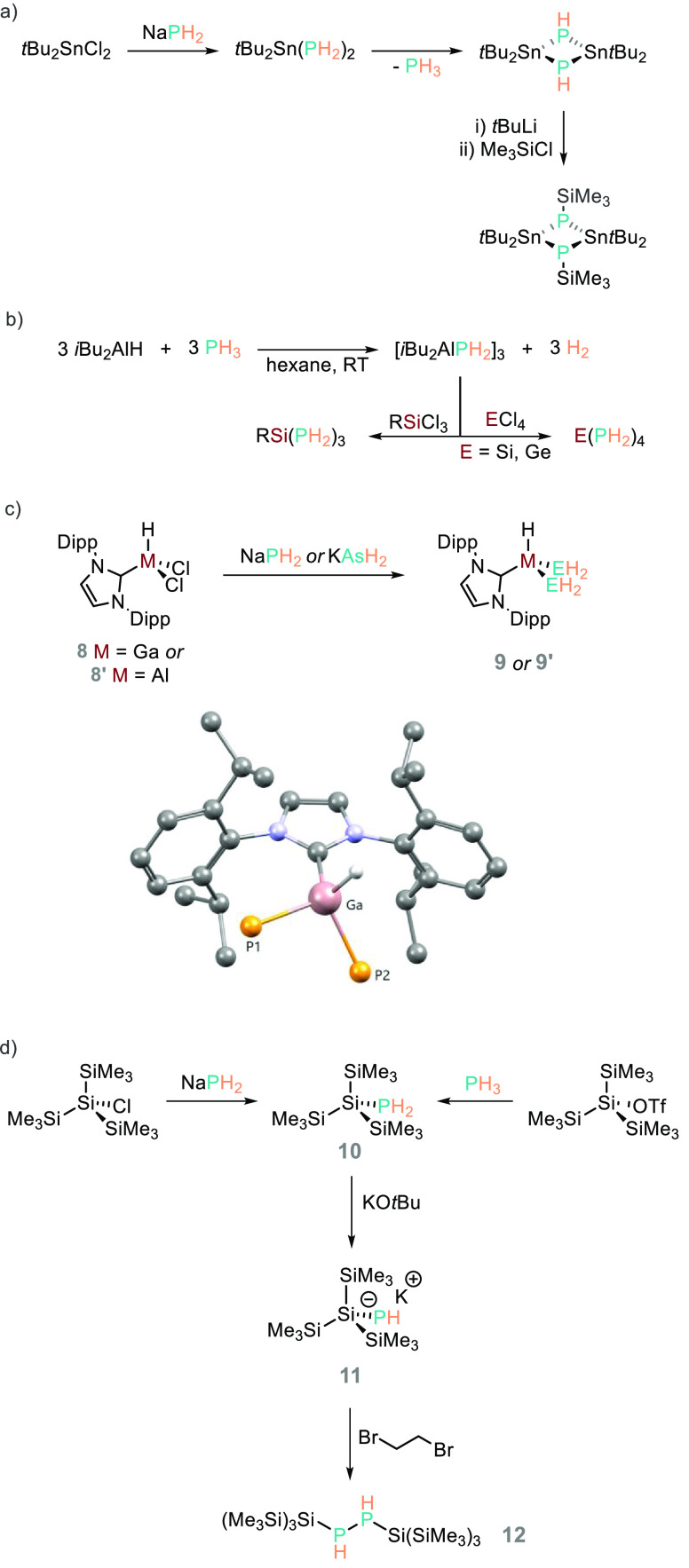
Various Main Group Bond Transformations Have Been Undertaken Using Metal Dihydrophosphides Including (a) the Formation of Sn–P Bonds; (b) Sn–PH_2_ and Ge–PH_2_ Compounds; (c) Al– and Ga–NHC Complexes Functionalized with PH_2_; (d) the Formation of Hypersilyl Substituted Phosphanes, Which Can Undergo Reductive Coupling, Forming **12** The Ga complex is depicted as the POV-Ray image (CCDC 2035403), with all H atoms, except the Ga–H fragment, removed for clarity.

Alkali metal sources of H_2_P^–^, *e.g*. LiPH_2_, NaPH_2_, KPH_2_, have been largely ignored in the literature until very recently, but given that they are prepared from PH_3_ and clearly have the potential to act as an alternative source of PH_3_ (*c.f*., Wolf), they are an intriguing reagent that deserves further investigation. Their limited use until now may be linked to the routes of synthesis and the instability of these MPH_2_ species. Joannis reported the first synthesis of Na and K dihydrogenphosphide in 1894;^77,78^ these compounds were further studied alongside the synthesis of LiPH_2_.^79−84^ Later still, the rubidium^85^ and cesium analogues were reported and the series of alkali metal dihydrogenphosphides were further characterized.^86^ These species were prepared by condensing PH_3_ in NH_3_(*l*) and reacting with the metal or metal amide. Handling the Li and Na adduct is not trivial; LiPH_2_ decomposes at room temperature while NaPH_2_ is noted to decompose above 393 K. The KPH_2_ and RbPH_2_ species are noted to decompose above 476 K, making them a robust reagent, and it is thus surprising that KPH_2_ has not been used more widely in the literature. The poor solubility of KPH_2_ can be improved by the addition of 18-crown-6 (catalytic quantities can be used) and/or the preparation of phthalimide anion adducts.^87^ It is worth noting that other routes to H_2_P^–^ anion adducts (e.g., phthalate, alkoxide complexes) are known.^88,89^ Several rudimentary transformations of MPH_2_ have been reported, where the products are often species that we could envisage as being useful building blocks ready for further reaction or functionalization. For example, Hänssgen reported the preparation of the planar 4-membered heterocycle [(*t*Bu_2_SnPH)_2_] from tBu_2_SnCl_2_ and NaPH_2_ (Scheme 14a).^90^ Driess has used a dehydrocoupling-type reaction to prepare a dihydrophosphido-aluminium compound [(*i*Bu_2_AlPH_2_)_3_] which also operates as an effective H_2_P^–^ transfer agent, forming tris(phosphane) or tetrakis(phosphaneyl)silane/germane products from the trichlorosilane or tetrachlorosilane/germane precursor (Scheme 14b).^91^ Scheer has undertaken salt metathesis reactions of NaPH_2_ with IPrGaHCl_2_ (**8**) and the Al analogue (**8′**) to generate the corresponding bis(dihydrophosphide) complexes (**9**/**9′**, Scheme 14c).^92^ Hassler undertook a study into hypersilyl substituted phosphanes and, as part of this investigation, employed PH_3_ or NaPH_2_ to prepare tris(trimethylsilyl)silyldihydrophosphide (**10**). This species can undergo deprotonation with KO*t*Bu, forming (Me_3_Si)_3_SiPHK (**11**), which is remarkably stable at room temperature, and can undergo reductive coupling to generate a mixture of the *meso*- and *rac*-isomers (**12**, Scheme 14d). This reductive coupling step involves reaction with *t*Bu_2_Hg or 1,2-dibromoethane; the latter indicates that the phosphide is not particularly nucleophilic in that a P–C bond is not formed between (Me_3_Si)_3_SiPHK and Br_2_(CH_2_)_2_.^93^ Again, this latter point is intriguing and could be further investigated.

**Scheme 15:**
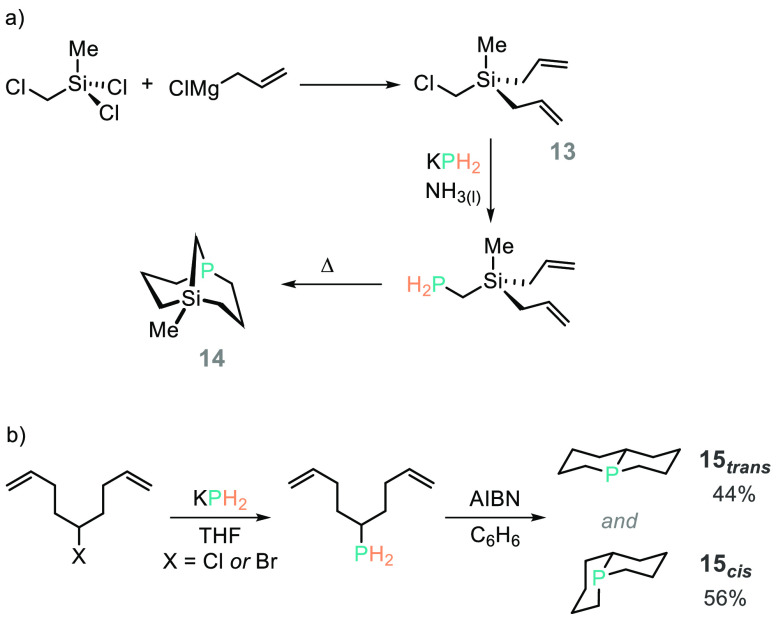
Issleib Has Used KPH_2_ To Install C–PH_2_ Bonds, Which Can Then Undergo Hydrophosphination To Generate Highly Unusual Bicycles

In 1982 Issleib reported on the use of KPH_2_ to form [3.3.1]-bicycle **14** by reaction with diallyl(chloromethyl)(methyl)silane (**13**) (Scheme 15a), and this type of protocol has since been used to access other phosphabicycles.^94,95^ A similar approach was taken to prepare a mixture of the *cis*- and *trans*-[4.4.0]-bicycle (**15**, Scheme 15b). These compounds were also complexed to Ni(CO)_4_, and the resulting L_*cis*_Ni(CO)_3_ and L_*trans*_Ni(CO)_3_ have similar Tolman Electronic Parameters (2063 and 2062 cm^–1^ respectively), which are very close to the σ-donor-only properties of PMe_3_ (2064 cm^–1^).^96^ Both reports indicate that interesting, unique phosphorus architectures can be prepared in a controlled way using PH_3_ derivatives.

**Scheme 16:**
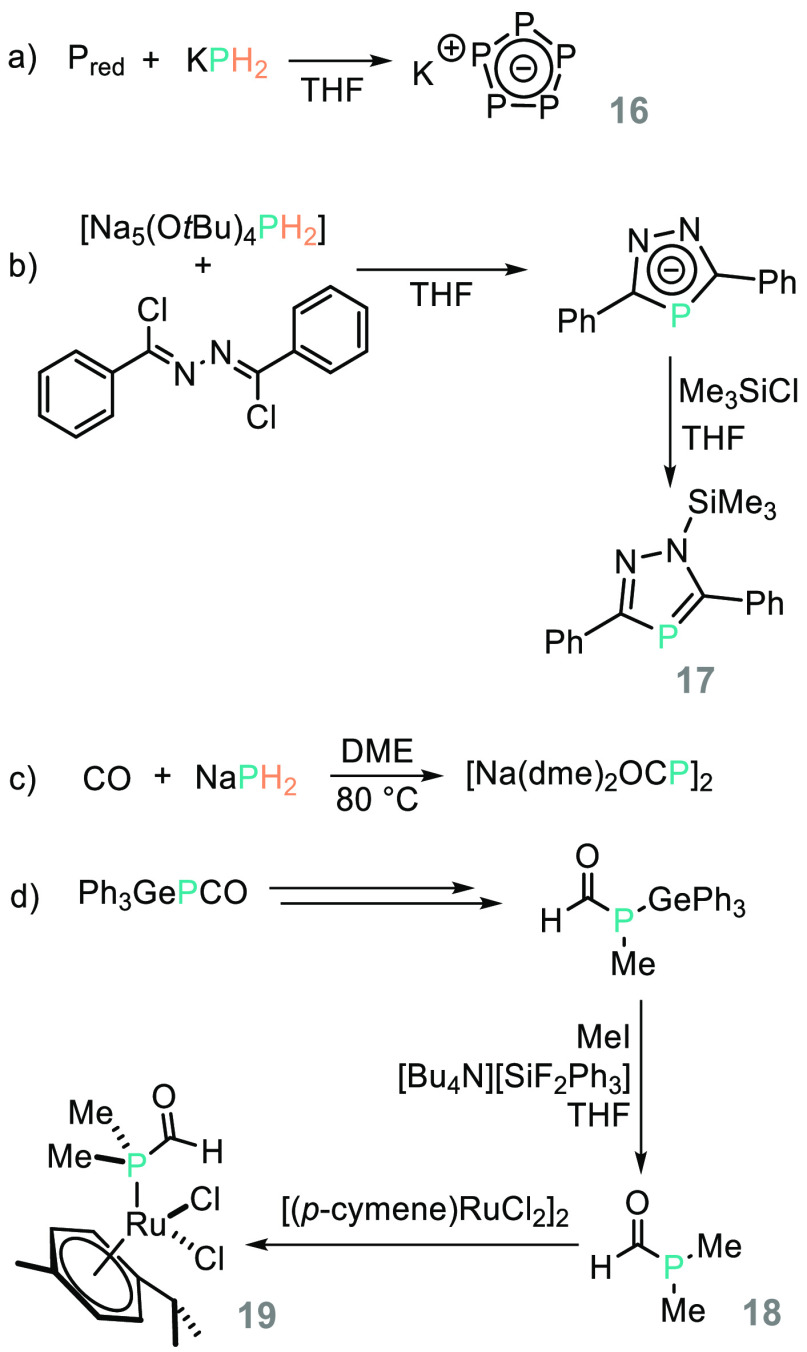
(a) An Improved Synthesis of Pentaphosphacyclopentadienide Was Achieved Using KPH_2_ in the Presence of Red Phosphorus; (b) Grützmacher and Co-workers Use Na_5_(O*t*Bu)_4_PH_2_ as a H_2_P^–^ Source to Prepare Elaborate Heterocycles; (c) Grützmacher’s Seminal Report on the Preparation of the Dme Adduct of NaOCP, Which Has Been Used to Great Effect in Main Group Synthesis (*vide infra*); (d) Stephan and Co-workers Prepare the Heavy Element Analogue of DMF and Demonstrate Elegant Coordination Chemistry of This Species

Baulder employed KPH_2_ in the degradation of red phosphorus to access the P_5_ anion, pentaphosphacyclopentadienide (the all-P analogue of the cyclopentadienyl anion) (**16**, Scheme 16a). With purification only requiring filtration and removal of PH_3_ gas, this offers an attractive alternative to the fractional crystallization previously reported for the synthesis from P_4_.^97^ Further to this example, use of NaPH_2_ (or Lewis base adduct analogues) is mostly limited to main group bond transformations. For example, Grützmacher reacted [Na_5_(O*t*Bu)_4_PH_2_] with 1,2-bis(chloro(phenyl)methylene)hydrazine to prepare a 1,2,4-diazaphospholide (**17**, Scheme 16b);^89^ the group also prepared the heavy isocyanate Na(OCP), sodium phosphaethynolate, from reaction of NaPH_2_ with CO (Scheme 16c).^98^ The onward reactivity of Na(OCP) (and Lewis base/solvent adducts) has been studied by Grüztmacher in terms of probing nucleophility in the presence of group 14 compounds,^99^ while Stephan has employed Gütztmacher’s germanium compound, Ph_3_GePCO, to prepare the phosphorus-containing analogue of *N*,*N*-dimethylformamide (**18**), which can undergo coordination chemistry with ruthenium forming **19** (Scheme 16d).^100^

### 7.2 Reactions of MPH_2_ with Carbonyl-Containing Compounds

**Scheme 17:**
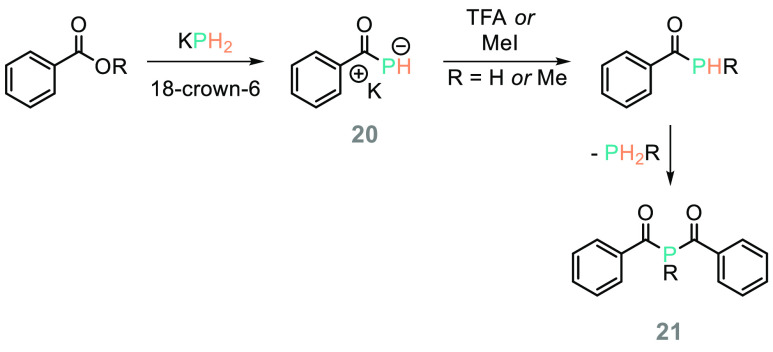
One of the Earliest Examples of Reactions of Carbonyl Compounds with KPH_2_ Was Presented by Liotta and Co-workers

An early report on potential applications in organic synthesis was provided by Liotta.^101^ Reaction of aryl or alkyl benzoates with KPH_2_ in the presence of a catalytic amount of 18-crown-6 (10 mol %) generates potassium benzoyl phosphide (**20**, Scheme 17). This can undergo protonation with acid (trifluoroacetic acid, TFA) or methylation with MeI, but in both cases the products are unstable and decompose to generate dibenzoylphosphines (**21**). As we might expect, based simply on atomic size, the authors note no partial double character due to P atom lone pair/carbonyl π-orbital overlap (as we normally see with amides); if decomposition pathways can be controlled it may be possible to develop useful chemistry that diverges from that of amides.

**Scheme 18:**
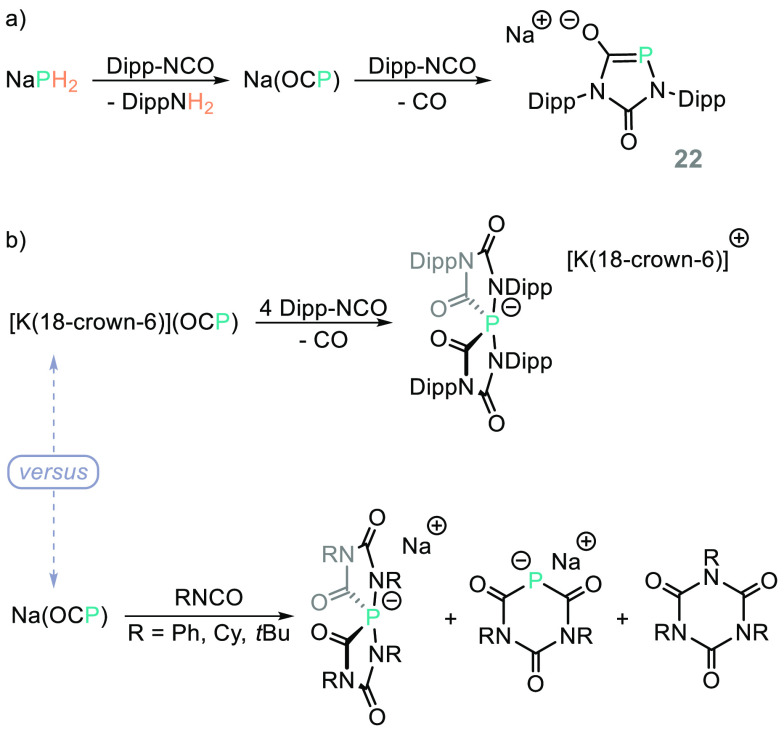
Reagent and Substrate-Dependent Activity Is Observed When Reacting Na(OCP) or [K(18-crown-6)](OCP) with Different Isocyanates

Goicoechea has reported the synthesis of Na(OCP) from the reaction of NaPH_2_ with isocyanate Dipp-NCO (though notably the syntheses of Na(OCP) have been reported from NaPH_2_ directly and PH_3_ as a feedstock).^102^ Na(OCP) goes on to react with isocyanates, generating structurally interesting main group compounds such as **22** (Scheme 18a).^103^ Interestingly, use of the potassium analogue, [K(18-crown-6)(OCP)], gives a different product distribution compared to that obtained using Na(OCP) (Scheme 18b), while the products obtained using Na(OCP) vary based on the substituents on the isocyanate (compare Scheme 18a and 18b, bottom),^104^ hinting at the diversity of synthesis that could be achieved if these reagents were more widely studied.

**Scheme 19:**
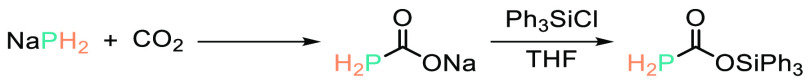
Goicoechea and Co-workers Prepare Silylesters from NaPH_2_ and CO

**Scheme 20:**
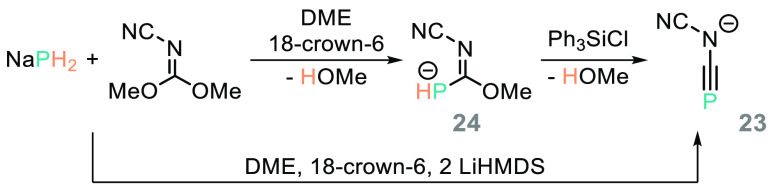
Goicoechea and Co-workers Demonstrate a Versatile Range of Main Group Transformations Using NaPH_2_ [Na(18-crown-6)]^+^ omitted for clarity.

Analogous to the work of Liotta on esters,^101^ reaction of NaPH_2_ with CO_2_ gives a phosphine carboxylate, which can then undergo onward reaction with silyl chlorides to form phosphine carboxylate silylesters (Scheme 19).^105^ Goicoechea has also shown that NaPH_2_ can react with dimethyl cyanocarbonimidate in one step to form the heteroallene anion species **23**, or in a stepwise fashion via the (carboximidate)phosphide **24** (Scheme 20). **24** can undergo reaction with Ph_3_GeCl to form a dimeric species product.^106^

Many of the reactions discussed thus far are rooted in fundamental research, and therefore for many of the reactions that could be termed transformations of main group species, it may be difficult to envisage the relevance of these compounds to the organophosphorus, organic chemistry or applied chemistry communities. While these main group compounds are often challenging to prepare and handle, organic transformations of carboxylic acids and allenes are well-known: we have yet to discover if the aforementioned phosphorus-containing species undergo the same transformations, *e.g*. allenes undergoing a rich array of addition and cyclization reactions.^107^

**Scheme 21:**
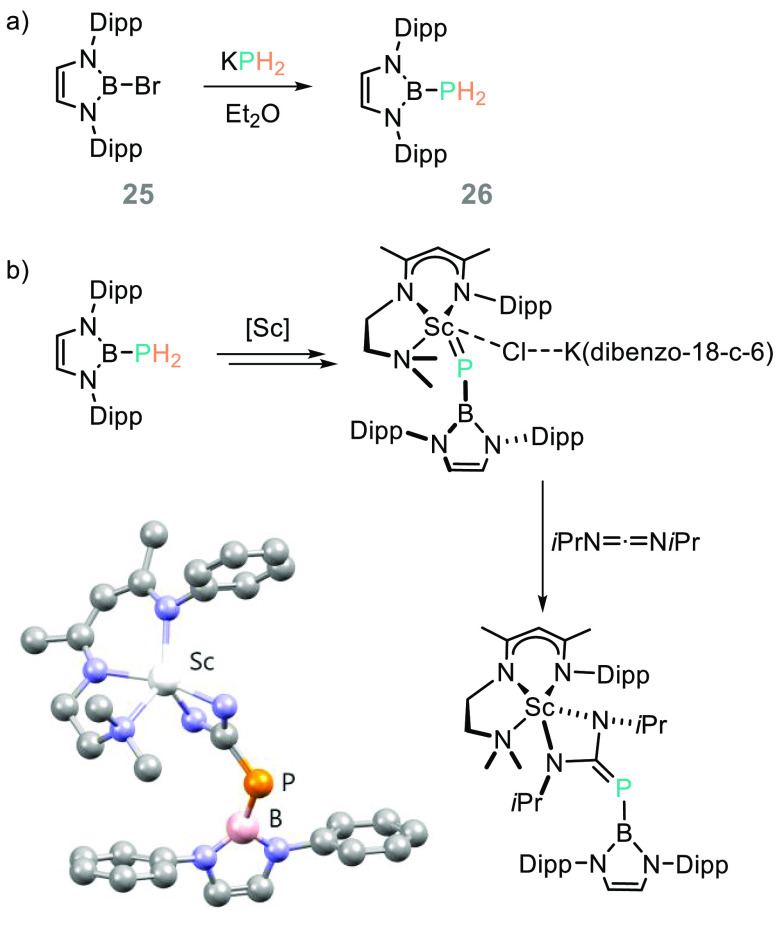
(a) Gudat and Co-workers Prepared and Studied the Electronic Properties of Phosphino-Diazaboroles, While (b) Chen, Maron and Co-workers Employed the System in Coordination Chemistry Dibenzo-18-crown-6 abbreviated for clarity (dibenzo-18-c-6). The Sc complex is depicted as the POV-Ray image (CCDC 2048477), with all iso-propyl groups and H atoms removed for clarity.

Gudat prepared a series of diazaboroles, including the PH_2_ species (**26**) from KPH_2_ and the bromodiazaborole precursor (**25**, Scheme 21a). The computational component of this study notes the covalent nature of the P–B σ-bond, along with the potential for these main group compounds to act as P-donor ligands.^108^ This ligand system has been incorporated into a scandium β-diketiminate complex,^109^ which can act as a phosphinidene transfer agent (Scheme 21b) similar to those already reported using a bulky 2,4,6-*t*Bu-phenyl phosphinidene Zr^110^ or Th^111^ complex, which operate in a stoichiometric fashion (akin to that possible with Tebbe’s reagent^112^).

### 7.3 Reactions of MPH_2_ with Compounds of the *d*- and *f*-Block

**Scheme 22:**
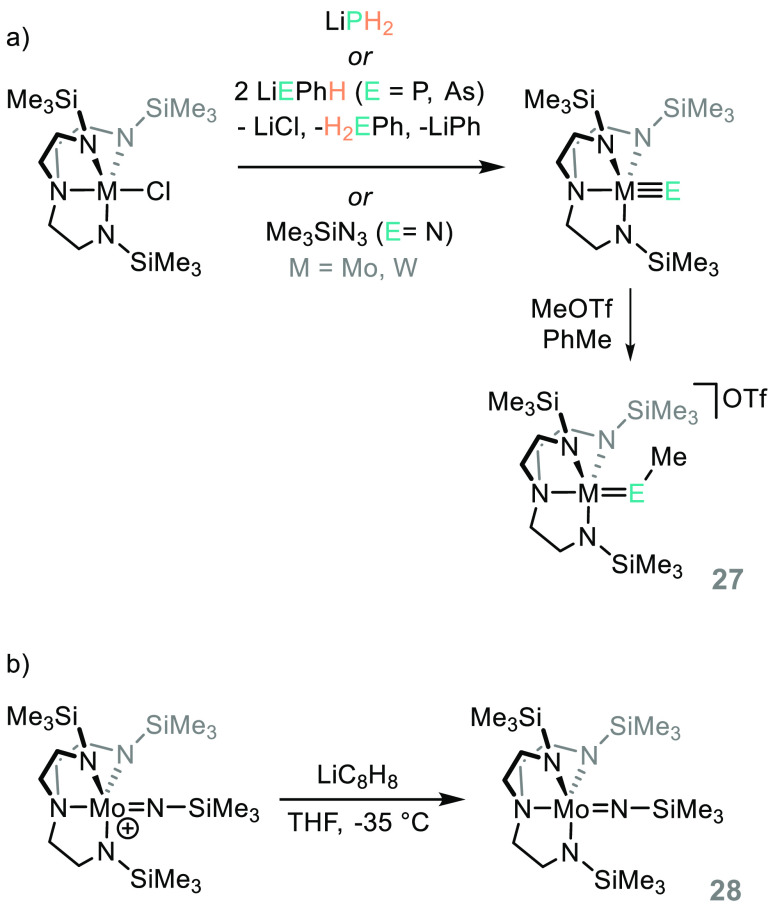
Schrock and Co-workers Have Undertaken a Systematic Study on the Coordination Chemistry of Molybdenum and Tungsten Amides, Phosphides, and Arsenides

Using a triamidoamine ligand, but one that is less bulky than that used to activate N_2_,^113^ Schrock was able to demonstrate the reactivity of a homologous series of Mo and W amido, phosphide, and arsenido complexes, employing LiPH_2_ or LiEPhH (E = P, As) or trimethylsilylazide (TMSN_3_) to install the M≡E bond (Scheme 22a).^114^ Their onward reaction with MeOTf to form the imido, phosphinidene, and arsinidene complexes (**27**) was studied, and the authors note that the Mo-phosphinidine complex decomposes in solution at room temperature whereas the W analogue does not. Similarly, the Mo arsinidine triflate was very unstable and could not undergo elemental analysis. The amido complex undergoes reduction in the presence of LiC_8_H_8_ to generate the Mo(V) product (**28**, Scheme 22b). This chemistry is important, because of not only the analogies we can draw between phosphorus and carbon but also the wealth of chemistry undertaken on the activation and functionalization of metal nitrido complexes, in particular their conversion to amines.^115−118^

**Scheme 23:**
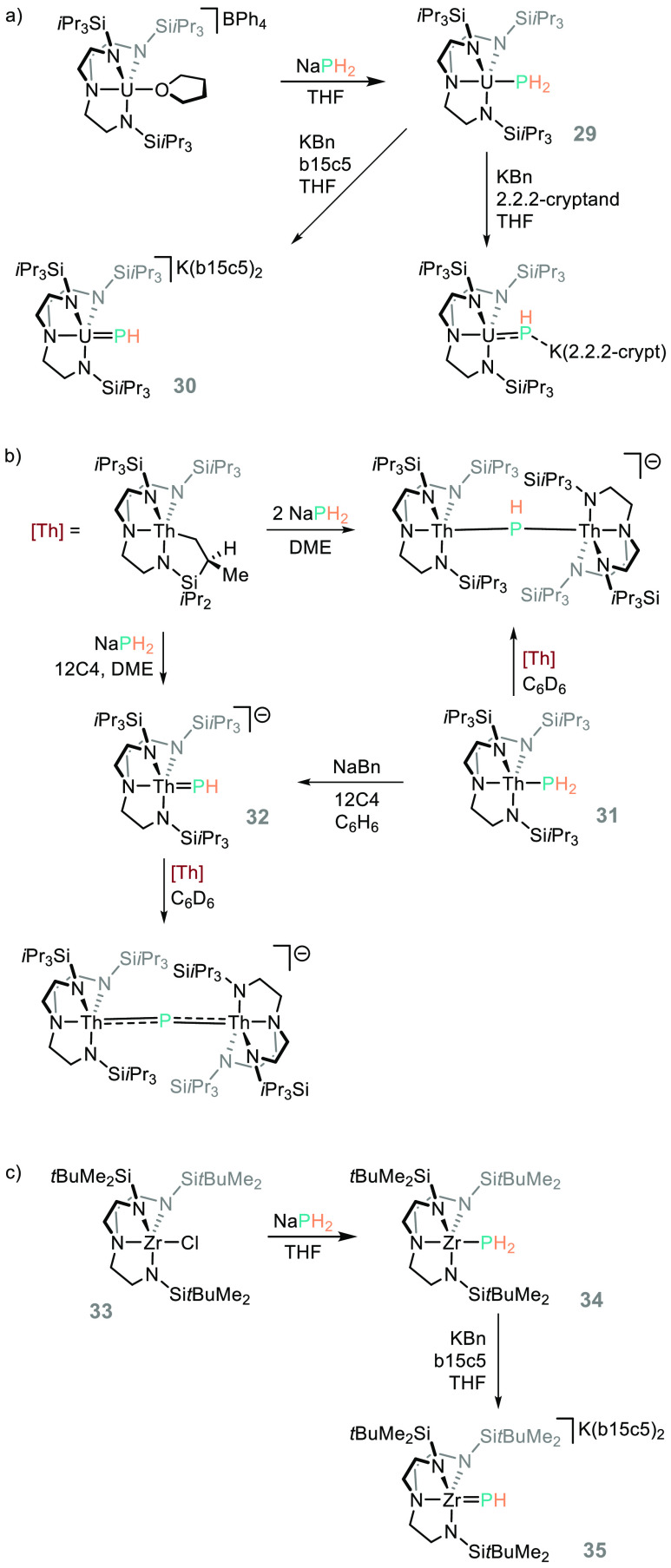
(a, b) Liddle’s Studies on the Phosphanide and Phosphinidene Chemistry of the Actinides; (c) Studies on the Analogous Zr Complex Na(12c4)_2_ cations omitted for clarity, and 12-crown-4 (12c4) and benzo-15-crown-5 abbreviated for clarity (b15c5).

If we consider the importance of metal–carbon double bonds in catalysis, *e.g*. in catalytic metathesis reactions, and the allegory between P and C, then it is vital that fundamental studies into bonding and reactivity of metal–P multiply bonded species are undertaken. In this regard, NaPH_2_ has been used by Liddle to generate uranium and thorium phosphanide (**29/31**) and phosphinidene (**30/32**) complexes (Scheme 23a and 23b).^119,120^ Liddle also presented a follow-up paper on the analogous Zr complex (**33**), which reacts in a similar way to the uranium and thorium analogues (forming the respective phosphanide and phosphinidene compounds, **34** and **35**, Scheme 23c).^121^ We can draw links to possible onward organic transformations by looking at the insertion chemistry reported by Stephan,^110^ Walter,^111^ and Walensky, where benzophenone was shown to insert into the Th–P bond of a bulkier mesityl analogue,^122^ and from the work of Mindiola on Ti-phosphinidene complexes and their hydrophosphination reactivity, although these species do require kinetic stabilization by use of a bulky organophosphine reagent.^123^

**Scheme 24:**
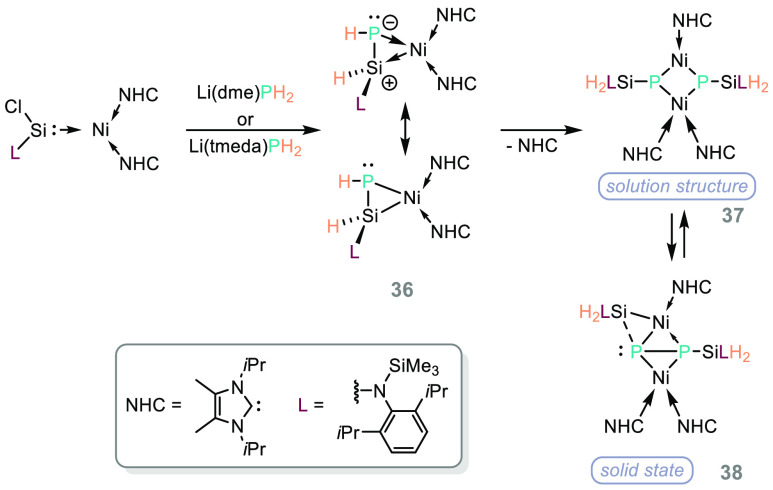
Driess and Co-workers Demonstrate the Versatility of Silylene Chemistry in Concert with Nickel NHCs

Driess has further elaborated the silylene chemistry of PH_3_^58^ by taking a nickel-silylene species and demonstrating the coordination chemistry of PH_2_ (derived from Li(dme)PH_2_ or Li(tmeda)PH_2_), generating **36** (Scheme 24) and subsequent isomerization chemistry of the η^2^-species to generate **37**/**38**.^124^

**Scheme 25:**
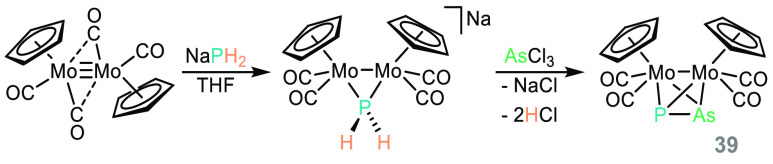
Scheer and Co-workers Prepare Mixed Group 15 Molybdenum Complexes Using NaPH_2_ (or LiE(SiMe_3_)_2_/KE(SiMe_3_)_2_, where E = P, As, Sb, Bi)

Finally, Scheer has also employed NaPH_2_ to generate a Mo dimer with a mixed P/As bridge (**39**, Scheme 25).^125^ A fundamental study, but one where we can envisage links to higher order main group polymer chains^126^ with unique properties.

## 8 Conclusions and Outlook

The utilization of PH_3_ in synthesis is undoubtedly an untapped well, and this is understandable owing to the significant challenges in manipulating pressurized cylinders of such a hazardous gas. However, the recent reports of *in situ* PH_3_ generation offer a much safer alternative to its traditional manipulation. These new operationally simple methods have the potential to revolutionize phosphorus research (which is itself prevalent across a wide range of disciplines). More readily accessible PH_3_ sources will also provide easier access to MPH_2_ species (where M is an alkali metal), which are already experiencing a renaissance and are proving vital to access novel phosphorus-containing species (*vide supra*).

Considerable efforts have gone into the functionalization of P_4_ and PCl_3_, and adding PH_3_ to the list of readily accessible phosphorus starting materials will grant access to a rich vein of research. The prospect of catalytically activating PH_3_ to access useful phosphorus reagents is exciting, and with reports of M=PH and M–PH_2_ species, this endeavor feels more attainable than ever. The question remains: how to take M=PH, M–PH_2_ and undertake transformations of these species that go beyond hydrophosphination chemistry reported in the 1980s and 1990s?
